# Increased levels of serum interleukin-10 are associated with poor outcome in adult hemophagocytic lymphohistiocytosis patients

**DOI:** 10.1186/s13023-021-01973-4

**Published:** 2021-08-04

**Authors:** Yulan Zhou, Fancong Kong, Shixuan Wang, Min Yu, Yawen Xu, Jing Kang, Songtao Tu, Fei Li

**Affiliations:** 1 Center of Hematology, The First Affiliated Hospital of Nanchang University, Nanchang, 330006 Jiangxi China; 2 Institute of Hematology, Academy of Clinical Medicine of Jiangxi Province, Nanchang, 330006 Jiangxi China

**Keywords:** Hemophagocytic lymphohistiocytosis, IL-10, Adult, Prognosis, Overall survival

## Abstract

**Background:**

Interleukin-10 (IL-10) is an independent factor for predicting adverse outcomes in pediatric patients with hemophagocytic lymphohistiocytosis (HLH). However, little is known about its prognostic value in adult patients.

**Methods:**

This single center retrospective study was conducted to explore the prognostic value of IL-10 in 101 adults newly diagnosed with HLH. The serum interleukin levels were quantitatively determined by chemiluminescence using cytokine profiling kits.

**Results:**

Serum IL-10 levels were significantly increased in adult HLH patients. Elevated IL-10 levels was correlated with lower concentrations of hemoglobin (r =  − 0.279, *P* = 0.005). IL-10 levels were significantly lower in patients with macrophage activation syndrome (MAS) than in those with infection-associated HLH (IAHS) and malignancy-associated HLH (MAHS) (*P* = 0.033, *P* = 0.012). Patients with MAS had relatively longer survival than those with IAHS and MAHS (*P* < 0.001). Univariate analysis indicated that hemoglobin < 8.2 g/dL, platelets < 40 × 10^9^/L, lactate dehydrogenase ≥ 700 IU/L, albumin < 28 g/L, post-treatment ferritin > 1050 µg/L and IL-10 ≥ 129 pg/mL were poor prognostic factors for survival. However, multivariate analysis revealed that only high serum IL-10 levels (≥ 129 pg/mL) at diagnosis and high post-treatment ferritin levels (> 1050 µg/L) were independent risk factors for poor overall survival in adult HLH patients (HR: 4.087, 95% CI 2.064–8.090, *P* < 0.001; HR 3.814, 95% CI 2.042–7.126, *P* < 0.001, respectively).

**Conclusions:**

Our results suggest that higher serum IL-10 levels might be a prognostic marker in adult HLH patients.

**Supplementary Information:**

The online version contains supplementary material available at 10.1186/s13023-021-01973-4.

## Introduction

Hemophagocytic lymphohistiocytosis (HLH, ORPHA: 158032) also known as hemophagocytic syndrome (HPS), is a rare and life-threatening hyperinflammatory syndrome characterized by pathologic immune activation [1, 2]. Although HLH is predominant among children, it has been seen in adults of all ages. Based on the etiology, HLH is generally classified into two types: primary HLH (pHLH, ORPHA: 158038) and secondary HLH (sHLH, ORPHA: 158041). The former is caused by inherited gene defects and mainly occurs in infants. sHLH is associated with various medical conditions, including persistent infection, malignancy and autoimmune disease, and mainly occurs in adults [3]. In patients with HLH, immune cells (such as T cells, NK cells and macrophages) are usually aberrantly activated, leading to over secretion of cytokines (a cytokine storm), including interferon-γ (IFN-γ), tumor necrosis factor-α (TNF-α), interleukin-6 (IL-6) and IL-10. Cytokines play an important role in HLH [4]. Cytokines are associated with several clinical features, including prolonged fever, hepatosplenomegaly, cytopenia, coagulopathy, liver damage and hyperferritinemia. IL-10 is an important T helper 2 (Th2) cytokine expressed by numerous cells of the innate and adaptive arms of the immune system, identified as a molecule that limits inflammation and supports humoral immune responses [5]. It regulates the balance between inflammatory and humoral responses [6]. IL-10 may increase the progression of HLH. Yang et al. [7] discovered that IL-10 may contribute to cytopenias in HLH. Luo et al. [8] found that an increased level of IL-10 at diagnosis was an independent prognostic factor for predicting adverse outcomes in HLH. While most studies have focused on pediatric HLH, the role of IL-10 in adult HLH has rarely been reported. In this retrospective study, 101 Chinese adults with HLH were evaluated to determine the patterns of IL-10 and elucidate the relationship between IL-10 and the clinical characteristics of adult patients with HLH.

## Patients and methods

### Patients

A total of 246 adult patients diagnosed with HLH between January 2015 and July 2020 at the First Affiliated Hospital of Nanchang University were reviewed retrospectively. The inclusion criteria for HLH patients were based on the HLH-2004 diagnostic guidelines [9]. Patients who had undergone cytokine measurement at diagnosis were considered. Demographic, clinical and laboratory data were recorded in all study participants. Exclusion criteria included exposure to immunosuppressive therapy before hospitalization and incomplete medical data. Follow-up was performed by reviewing medical records or making phone calls to the patients and their next of kin. The overall survival (OS) was estimated from the date of diagnosis to the date of death or last follow-up (July 5, 2020).

### Detection of cytokines

Serum samples that were collected at diagnosis were used for the measurement of serum cytokines. The levels of IL-1β, IL-8 and IL-10 were measured with cytokine profiling kits (Siemens Healthcare (Pty) Ltd.) using the Immulite® 1000 Immunoassay System (Siemens Healthcare (Pty) Ltd) according to the manufacturer’s instructions.

### Statistical analysis

Numerical data were expressed as mean ± SD or median with interquartile range. Categorical variables were expressed as numbers and percentages. Continuous variables were compared using the t test or Mann–Whitney U tests. Categorical variables were analyzed using the chi-square test or Fisher’s exact test. Cytokines and routine laboratory parameters were defined as dichotomous variables based on median as the cut-off point according to previous studies [10]. The correlation between laboratory tests and various cytokines was determined by Spearman’s rank correlation analysis. Survival outcomes were estimated using the Kaplan–Meier method and compared with the log-rank test. Multivariate Cox analysis (hazard ratios (HR) and 95% CI) was used to identify the independent prognostic variables for OS. A *P* value < 0.05 was considered statistically significant. Analyses were performed using SPSS software (version 24).

## Results

### Characteristics of the enrolled participants

**Fig. 1:**
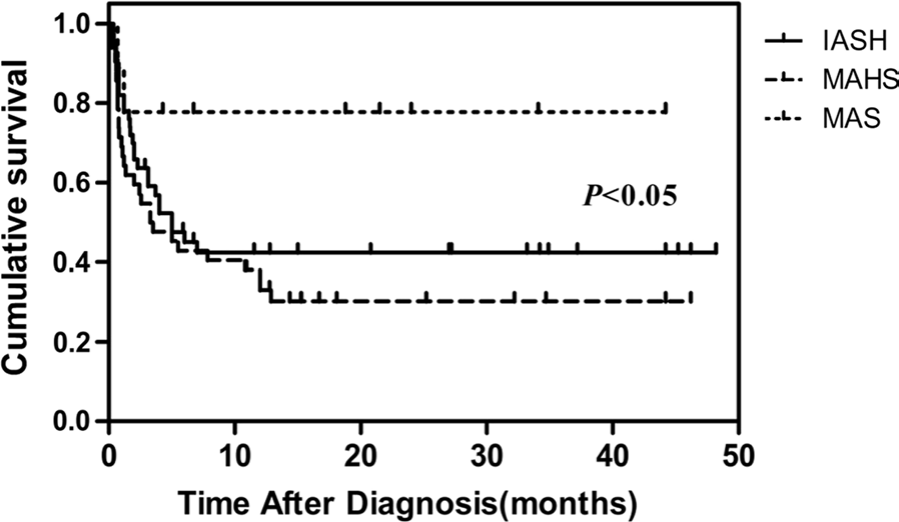
Survival outcomes in adult secondary HLH patients. Kaplan–Meier curve showing the overall survival (OS) according to the cause of secondary HLH. Patients with macrophage activation syndrome (MAS) had the longer median survival time than those with infection-associated HLH (IAHS) and malignancy-associated HLH (MAHS) (*P* < 0.05)

Of 246 adult patients with HLH, 101 (55 males, 46 females) met the inclusion criteria for analysis. The median age was 49 years (range 18–89 years). Among the enrolled patients were 50 with infection-associated HLH (IAHS), 42 with malignancy-associated HLH (MAHS) and 9 with macrophage activation syndrome (MAS) (Additional file 1: Fig. 1). Patients in this cohort received the HLH-94/04 regimen, DEP regimen (doxorubicin-etoposide-methylprednisolone), or simple glucocorticoid therapy to control their symptoms. They received the treatment protocol best suited for the etiology of the HLH and supportive care. Epstein-Barr virus (EBV) was the most frequent etiology in IAHS patients (37/50, 74.0%). Most MAHS patients presented with lymphoma (38/42, 90.5%). The median follow-up time was 4.0 months (range 0.2–48.2 months). At the time of the last follow-up (July 5, 2020), 58 patients (57.4%) had died. Comparisons of demographic and clinical characteristics between HLH patients who survived and those who died are shown in Additional file 1: Table 1. Neutrophil counts, platelet counts and hemoglobin levels were significantly lower in patients who died compared to those who survived. The median OS of the entire cohort was 5.0 months. There were significant differences in prognosis among the three groups. Patients with MAS had relatively longer survival compared to those with IAHS and MAHS. Patients with MAHS had the shortest median survival (3.4 months, Fig. 1).

### Clinical manifestations and laboratory findings

All patients presented with a history of one week to two months of high-grade fever (median 21 days, range 7 to 82 days), with temperature fluctuating from 38.5 to 41.0 °C. Common clinical features of HLH included splenomegaly (86/101, 85.14%), hepatomegaly (28/101, 27.7%) and lymphadenopathy (67/101, 66.3%). Cytopenias (affecting at least 2 of 3 lineages in the peripheral blood) were found in 58.4% (59/101) of patients. Neutropenia (< 1.0 × 10^9^/L), anemia (< 90 g/L) and thrombocytopenia (< 100 × 10^9^/L) occurred in 53.5% (54/101), 57.4% (58/101), and 82.2% (83/101) of patients, respectively. Most patients presented with abnormal liver function: elevated aspartate aminotransferase (AST) (> 50 U/L, 70/101, 69.3%), alanine aminotransferase (ALT) (> 50 U/L, 73/101, 72.3%), total bilirubin (> 17.1 mmol/L, 43/101, 42.6%) and lactate dehydrogenase (LDH) (> 300 IU/L, 93/101, 92.1%). Hyperferritinemia (≥ 500 μg/L) was documented in all patients, with 77.2% (78/101) of patients having a ferritin concentration higher than 2000 ng/mL. Other laboratory findings included hypofibrinogenemia (< 1.5 g/L, 40/101, 39.6%), hypertriglyceridemia (> 3 mmol/L, 36/101, 35.6%), prolonged activated partial thromboplastin time (10 s greater than control) (45/101, 44.6%), prolonged prothrombin time (3 s greater than control) (16/101,15.8%), elevated soluble CD25 (> 2400 U/mL, 87/98, 88.8%) and hemophagocytosis in the bone marrow (57/97, 58.8%).

### Cytokines

**Fig. 2:**
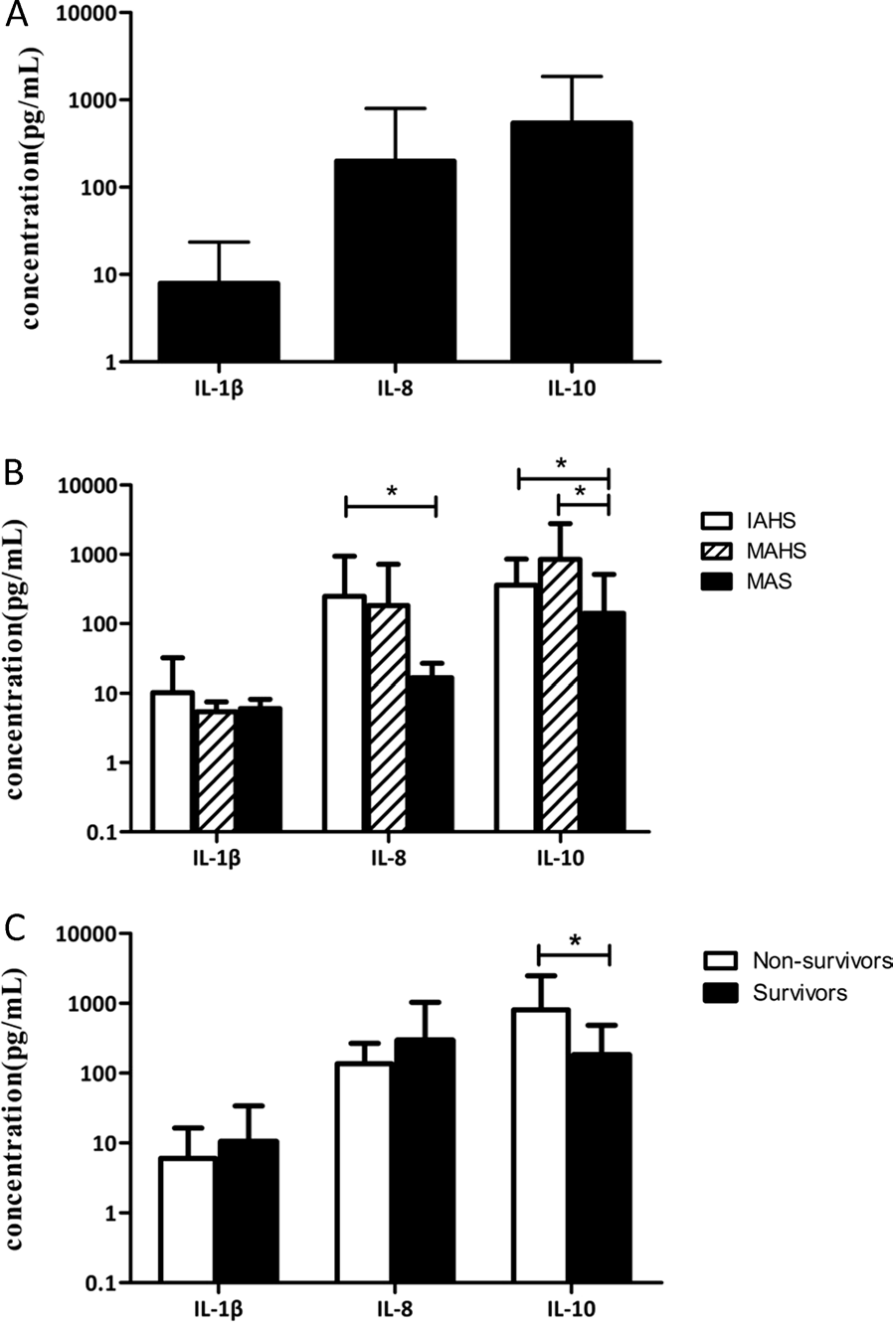
Cytokine levels in HLH. **A** Expression levels of IL-1β, IL-8 and IL-10 in HLH. **B** IL-8 levels were significantly higher in IAHS than in MAS (p = 0.024), IL-10 levels were significantly higher in IAHS and MAHS compared with MAS (*P* = 0.033, *P* = 0.012). **C** IL-10 levels were significantly elevated in non-survivors compare with survivors (*P* = 0.007)

As shown Additional file 1: Table 2 and Fig. 2A, the levels of IL-8 and IL-10 were significantly elevated in most patients. IL-1β was almost in the normal range. IL-10 levels were significantly higher in IAHS and MAHS patients compared with MAS patients (*P* = 0.033, *P* = 0.012). IL-8 levels were significantly higher in IAHS patients than in MAS patients (*P* = 0.024). However, there was no significant difference in IL-1β levels among the three groups (Fig. 2B). Moreover, IL-10 levels were significantly higher in the group that died (*P* = 0.007, Fig. 2C).

### Relationship between cytokines and routine laboratory parameters

We investigated the relationship between serum IL-10 levels and various laboratory parameters such as the full blood count, liver function and ferritin. Serum IL-10 levels were significantly negatively correlated with the concentrations of hemoglobin (r =  − 0.279, *P* = 0.005, Table 1). IL-10 levels were significantly higher in HLH patients who had cytopenia of two or more lineages than in those who did not (*P* = 0.026).

**Table 1:** Relations between IL-10 and clinical parameters in HLH

Parameters	Correlation coefficient	*P*
Neutrophil	− 0.063	0.533
Hemoglobin	− 0.279	0.005*
Platelet	− 0.113	0.261
Fibrinogen	0.119	0.238
Triglyceride	− 0.031	0.760
Lactate dehydrase	− 0.053	0.600
Albumin	− 0.182	0.068
Ferritin	− 0.095	0.347
IL-1β	− 0.067	0.507
IL-8	− 0.036	0.725

*Indicates statistically significant values (*P* < 0.05)

### Relationship between cytokines and mortality

**Fig. 3:**
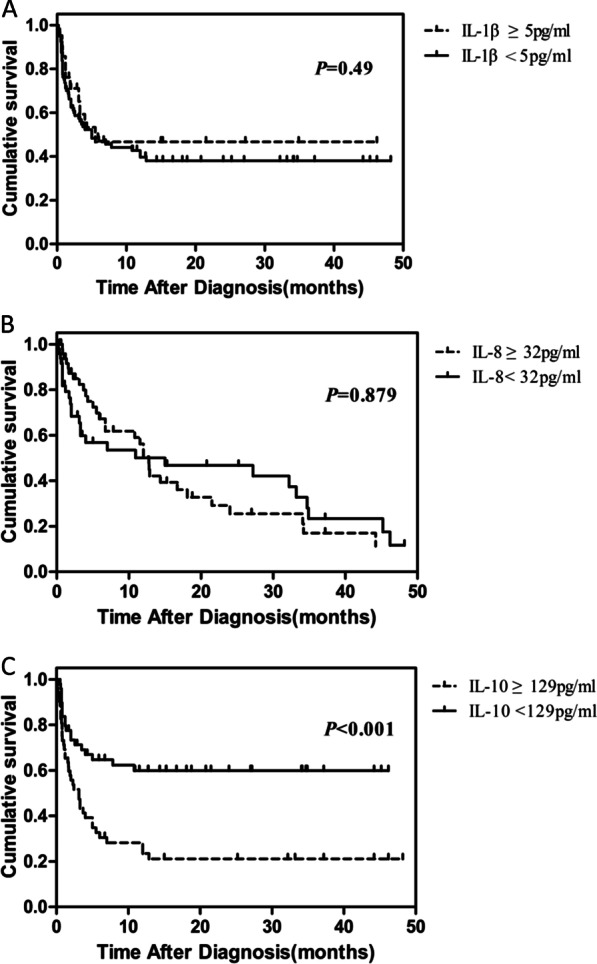
Survival outcomes based on the serum cytokine level. Kaplan–Meier curve showing the OS based on the levels of IL-1β, IL-8 and IL-10. **A** No statistically significant difference was observed in OS between HLH patients with IL-1β < 5 pg/mL and those with IL-1β ≥ 5 pg/mL (*P* = 0.49). **B** No statistically significant difference was observed in OS between HLH patients with IL-8 < 32 pg/mL and those with IL-8 ≥ 32 pg/mL (*P* = 0.879). **C** HLH patients with serum IL-10 ≥ 129 pg/mL showed significantly worse OS compared to those with serum IL-10 < 129 pg/mL (*P* < 0.001)

Univariate and multivariate analyses were conducted to identify possible relationships between IL-10 and mortality. The cut-off values of cytokines and routine laboratory parameters were determined by their median values. Median OS was significantly shorter in HLH patients with IL-10 ≥ 129 pg/mL (3.12 months) than in those with IL-10 < 129 pg/mL (not reached half deaths, *P* < 0.001, Fig. 3C). However, no significant difference in OS was observed between groups for IL-1β (Fig. 3A) and IL-8 (Fig. 3B). The Kaplan–Meier survival analysis showed that patients with IAHS or MAHS and high serum IL-10 levels had poor OS (*P* < 0.001, *P* = 0.001, respectively, Additional file 1: Fig. 2). In univariate survival analysis, hemoglobin < 8.2 g/dL, platelet < 40 × 10^9^/L, albumin < 28 g/L, LDH ≥ 700 U/L, post-treatment ferritin > 1050 µg/L and IL-10 ≥ 129 pg/mL were associated with a worse outcome (*P* = 0.028; *P* = 0.026; *P* = 0.019; *P* = 0.045; *P* < 0.001, *P* < 0.001, respectively, Table 2). However, in the multivariate Cox model, only IL-10 and post-treatment ferritin were independent predictors of poor OS (*P* < 0.001; *P* < 0.001, respectively, Table 3).

**Table 2:** Univariate analysis of risk factors for OS in newly diagnosed adult HLH patients

Prognostic factor	Overall		
	HR	95%CI	*P*
Hemoglobin < 8.2 g/dL	1.853	1.069, 3.213	0.028*
Platelets < 40 × 10^9^/L	1.798	1.072, 3.015	0.026*
Fibrinogen < 2.0 g/L	0.871	0.520, 1.458	0.599
LDH ≥ 700 IU/L	1.711	1.012, 2.891	0.045*
Albumin < 28 g/L	2.341	1.148, 4.775	0.019*
Triglycerides > 220 mg/dL	0.997	0.571, 1.741	0.993
Ferritin > 2000 µg/L	0.663	0.371, 1.184	0.165
Post-treatment ferritin > 1050 µg/L	4.964	2.760, 8.926	< 0.001*
IL-1β ≥ 5 pg/mL	1.327	0.650, 2.705	0.437
IL-8 ≥ 32 pg/mL	1.312	0.780, 2.207	0.307
IL-10 ≥ 129 pg/mL	6.736	3.567, 12.721	< 0.001*

HR, hazard ratio; CI, confidence interval

*Indicates statistically significant values

**Table 3:** Multivariate analysis of risk factors for OS in newly diagnosed adult HLH patients

Prognostic factor	HR	95% CI	*P*
Hemoglobin < 8.2 g/dL	1.100	0.612, 1.978	0.750
Platelets < 40 × 10^9^/L	1.589	0.914, 2.764	0.101
LDH ≥ 700 IU/L	0.880	0.458, 1.692	0.702
Albumin < 28 g/L	1.625	0.779, 3.390	0.196
Post-treatment ferritin > 1050 µg/L	3.814	2.042, 7.126	< 0.001*
IL-10 ≥ 129 pg/mL	4.087	2.064, 8.090	< 0.001*

HR, hazard ratio; CI, confidence interval

*Indicates statistically significant values

## Discussion

IL-10 is a pleomorphic cytokine with diverse phenotypic functions [11]. The expression of IL-10 is altered in numerous human diseases including cancer, autoimmune diseases and inflammatory diseases [12–14]. Osugi et al. and An et al. [15, 16] showed that serum concentrations of IL-10 increased in children with HLH and that IL-10 plays a critical role in the pathogenesis of HLH. However, the role of IL-10 in adult HLH remains unknown. The etiologic conditions associated with HLH in our cohort included rheumatic diseases in 9 patients, infections in 50 patients and malignancies in 42 patients. The most frequent triggers in our cohort were infections (49.5%) and malignancies (41.6%), which were consistent with other adult HLH cohorts that reported underlying diseases [2, 17, 18]. We demonstrated elevated serum levels of IL-10 in adult HLH patients. IL-10 levels were significantly higher in IAHS and MAHS patients compared with MAS patients. A recent study by Heper et al. [19] found that systemic levels of IL-10 were useful in predicting mortality in patients with sepsis. IL-10 levels were higher in patients who died compared to those who survived. Previous studies demonstrated that patients with MAHS and IAHS had a poorer outcome compared to patients with MAS [17, 20, 21]. Similar results were also observed in the present study where patients with MAS had a better outcome compared to those with other triggering conditions.

Correlation analysis revealed that IL-10 levels were closely correlated with hemoglobin levels (*P* = 0.005). In addition, IL-10 levels were significantly higher in HLH patients who had cytopenia of two or more lineages compared to those who did not. These results were similar to those of Yang et al. [7] who found that IL-10 might contribute to cytopenias in pediatric patients with HLH.

Previous studies have found that adverse outcomes are more common in HLH patients with certain pretreatment laboratory findings. A previous study in pediatric patients with secondary HLH found that severe hypoalbuminemia was an independent risk factor for 30-day mortality, and other biochemical parameters, including hemoglobin, fibrinogen and ferritin levels, did not influence the risk of death [22]. In another study involving 116 children with secondary HLH, Qiong et al. [23] found that low neutrophil count, low albumin level, high bilirubin level and high LDH level were significantly associated with a high risk of early death due to HLH. However, variables such as hemoglobin level, platelet count, fibrinogen level, triglyceride level and ferritin level were not significantly associated with early adverse outcomes. Sameer et al. [24] investigated adults with secondary HLH and found that high LDH levels, low albumin levels and high ferritin levels were associated with a worse prognosis. Anna et al. [25] found that various parameters, including low platelet counts, low hemoglobin levels, high alanine aminotransferase levels, high bilirubin levels, low albumin levels, high ferritin levels and low fibrinogen levels, were predictive of survival outcomes in adults with non-malignancy associated secondary HLH. Moreover, Zhou et al. [26] found that post-treatment serum ferritin ≥ 1050 µg/L may serve as an independent prognostic biomarker in adult HLH patients. Similar to previous studies, we found that low hemoglobin level (< 8.2 g/dL), low platelet counts (< 40 × 10^9^/L), low albumin concentrations (< 28 g/L), high LDH levels (≥ 700 U/L) and high post-treatment serum ferritin levels (≥ 1050 µg/L) were associated with poor survival.

Cytokines have been shown to play important roles in the development of HLH [4]. Some cytokines were found to be correlated with prognosis of HLH. In a retrospective study of 155 patients, Lu et al. [27] demonstrated that IL-6, which contributes to acute systemic inflammatory response syndrome [28], was an independent negative predictor of HLH. Recent studies have indicated that IL-10 levels may be associated with adverse outcomes in pediatric HLH. Tang et al. [29] showed that significantly increased IL-10 was an early, specific and adverse prognostic risk factor for childhood HLH. Luo et al. [8] found that elevated IL-10 levels at diagnosis were independent prognostic factors for predicting death in children with HLH. Earlier this year, based on a small-sample study, Li et al. [30] showed that IL-10 was an important risk factor for early death in patients with sHLH. More interesting, they reported that high levels of IL-10 (≥ 16.73 pg/mL) predicted worse prognosis of sHLH. In the present study, we found that the level of IL-10 was higher in patients with poor survival. In addition, patients with IL-10 < 129 pg/mL had longer OS compared to those with IL-10 ≥ 129 pg/mL regardless of the underlying etiologies, suggesting that high IL-10 levels were associated with mortality in adult HLH patients. Furthermore, a high serum IL-10 level was an independent risk factor for poor OS in adult HLH patients. However, unlike post-treatment serum ferritin, serum IL-10 levels can be used for prognostic evaluation before therapy.

It is well known that IL-10 mainly acts as a negative immune regulator in numerous diseases [11], as it inhibits the production of inflammatory mediators [31]. Our data suggest a possible role of IL-10 as an independent prognostic biomarker in adult HLH patients. Because HLH is a hyperinflammatory syndrome, we hypothesize that a regulatory feedback loop exists through which pro-inflammatory cytokines upregulate the anti-inflammatory cytokine IL-10 in HLH. Therefore, IL-10 may act as an ‘alarm hormone’, which reflects disease severity in HLH.

There are several limitations in the present study. First, it is a single center, retrospective cohort study. There were missing clinical data and patients who were lost to follow-up. Second, the study does not include cytokines such as IL-6, TNF-α and IFN-γ, which may also be potential prognostic indicators in HLH. Third, the relatively small number of patients might introduce a bias in our results. Therefore, further prospective multicentre studies with larger sample sizes are needed to validate our findings.

## Conclusions

Our study suggests that elevated IL-10 (≥ 129 pg/mL) at diagnosis may be used as an independent prognostic marker in adult HLH patients to guide treatment strategies.

## Supplementary Information

**Additional file 1: Table S1.** Demographic, laboratory data and HLH manifestations. **Table S2**. The expressions of cytokines in patients with HLH. **Figure S1**. Flowchart of patient recruitment and classification. **Figure S2**. Performance of serum IL-10 in patients with various etiologies. (A). Infection-associated HLH patients with high serum IL-10 levels showed significantly worse OS than those with low serum IL-10 levels (*P* < 0.001). (B). Malignancy-associated HLH patients with high serum IL-10 levels showed significantly worse OS than those with low serum IL-10 levels (*P* = 0.001)

## Data Availability

The data used to support the findings of this study are available from the corresponding author upon request.
