# Supplementary material for: Increased levels of serum interleukin-10 are associated with poor outcome in adult hemophagocytic lymphohistiocytosis patients
Source: Orphanet J Rare Dis. 2021 Aug 4;16:347. doi: 10.1186/s13023-021-01973-4 (PMC8336343; doi:10.1186/s13023-021-01973-4)
Supplement: Supplementary file 1 — Additional file 1: Table S1. Demographic, laboratory data and HLH manifestations. Table S2. The expressions of cytokines in patients with HLH. Figure S1. Flowchart of patient recruitment and classification. Figure S2. Performance of serum IL-10 in patients with various etiologies. (A). Infection-associated HLH patients with high serum IL-10 levels showed significantly worse OS than those with low serum IL-10 levels (P < 0.001). (B). Malignancy-associated HLH patients with high serum IL-10 levels showed significantly worse OS than those with low serum IL-10 levels (P = 0.001) [file 13023_2021_1973_MOESM1_ESM.docx]

**Supplementary Table 1. Demographic, laboratory data and HLH manifestations**

| Characteristics | Alive | Dead | t/χ2 | P |
| --- | --- | --- | --- | --- |
| Age (years) | 41.86±16.29 | 48.29±17.81 | 1.742 | 0.085 |
| Males (n, %) | 16 (45.71%) | 35 (60.34%) | 1.887 | 0.170 |
| Splenomegaly (n, %) | 31 (88.57%) | 48 (82.76%) | 0.577 | 0.448 |
| Hypertriglyceridemia (n, %) | 8 (22.86%) | 23 (39.66%) | 2.772 | 0.096 |
| Cytopenia (at least two lineages) (n, %) | 11 (31.43%) | 45 (77.59%) | 19.412 | <0.001* |
| Fibrinogenemia <1.5g/L (n, %) | 15 (42.86%) | 22 (37.93%) | 0.221 | 0.638 |
| sCD25≥2400U/mL (n, %) | 29 (82.86%) | 53 (91.38%) | 1.520 | 0.218 |
| Hemophagocytosis in bone marrow (n, %) | 22 (62.86%) | 33 (56.90%) | 0.321 | 0.571 |
| Neutrophil | 4.31±4.44 | 2.36±1.79 | -2.97 | 0.004* |
| Hemoglobin | 90.89±15.87 | 82.45±21.81 | -1.991 | 0.049* |
| Platelet | 85.06±72.10 | 49.34±45.72 | -2.629 | 0.011* |
| Fibrinogen | 2.47±1.88 | 2.31±1.50 | -0.450 | 0.654 |
| Triglyceride | 2.43±1.79 | 2.93±1.40 | 1.499 | 0.137 |
| Albumin | 28.80±4.29 | 28.24±5.77 | -0.496 | 0.621 |

**Supplementary Table 2. The expressions of cytokines in patients with HLH**

| Variables | HLH(median and range, pg/mL) | Upper limit of normal (pg/mL) |
| --- | --- | --- |
| IL-1β | 5 (1, 159) | 5 |
| IL-8 | 32 (2, 3452) | 62 |
| IL-10 | 129 (2, 10000) | 9 |

**Supplementary Figure 1**

**
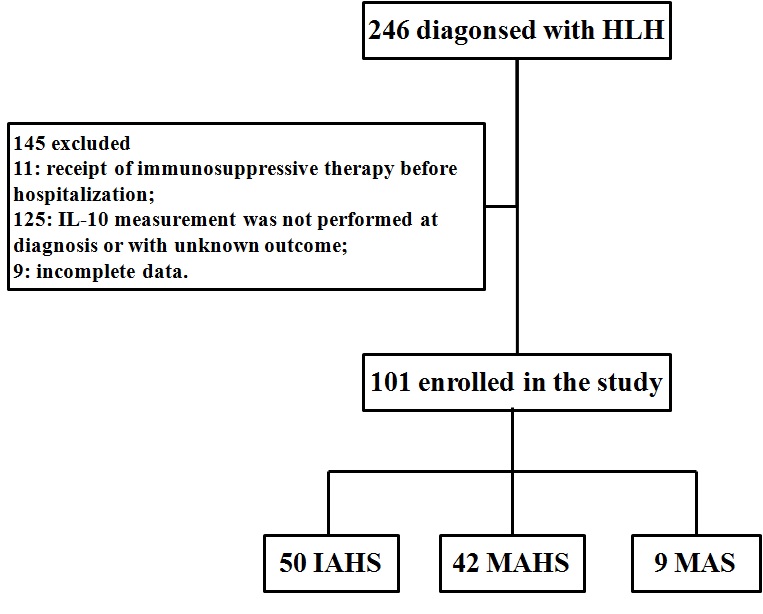
**

**Supplementary Figure1. Flowchart of patient recruitment and classification.**

**Supplementary Figure 2A**


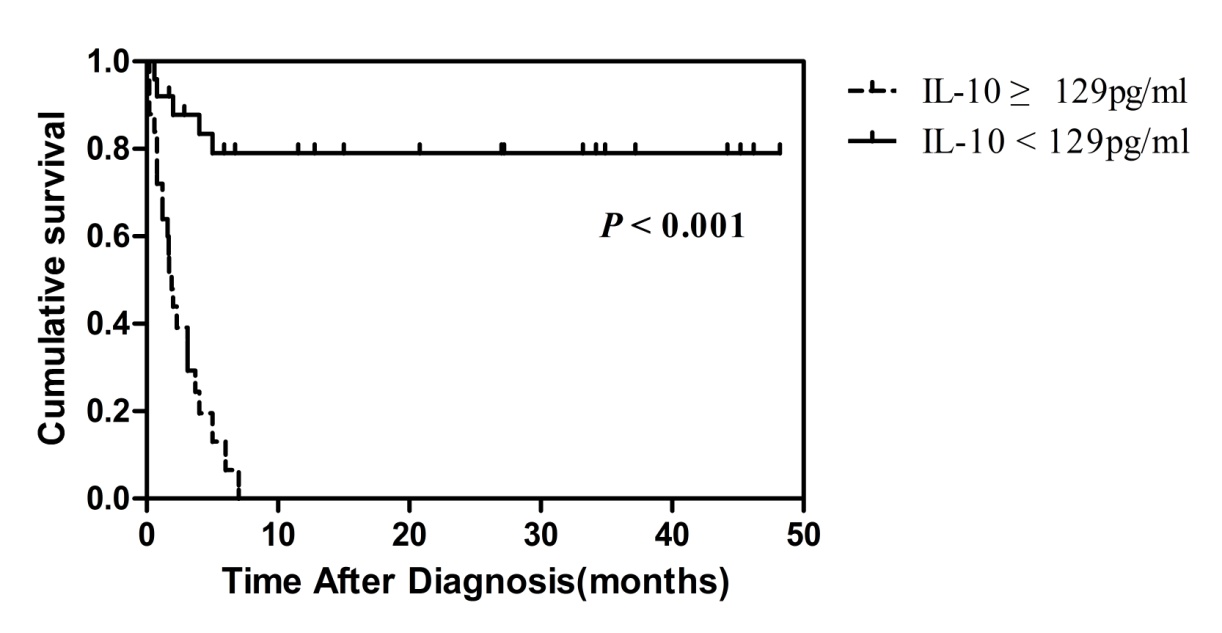


**Supplementary Figure 2B**

**
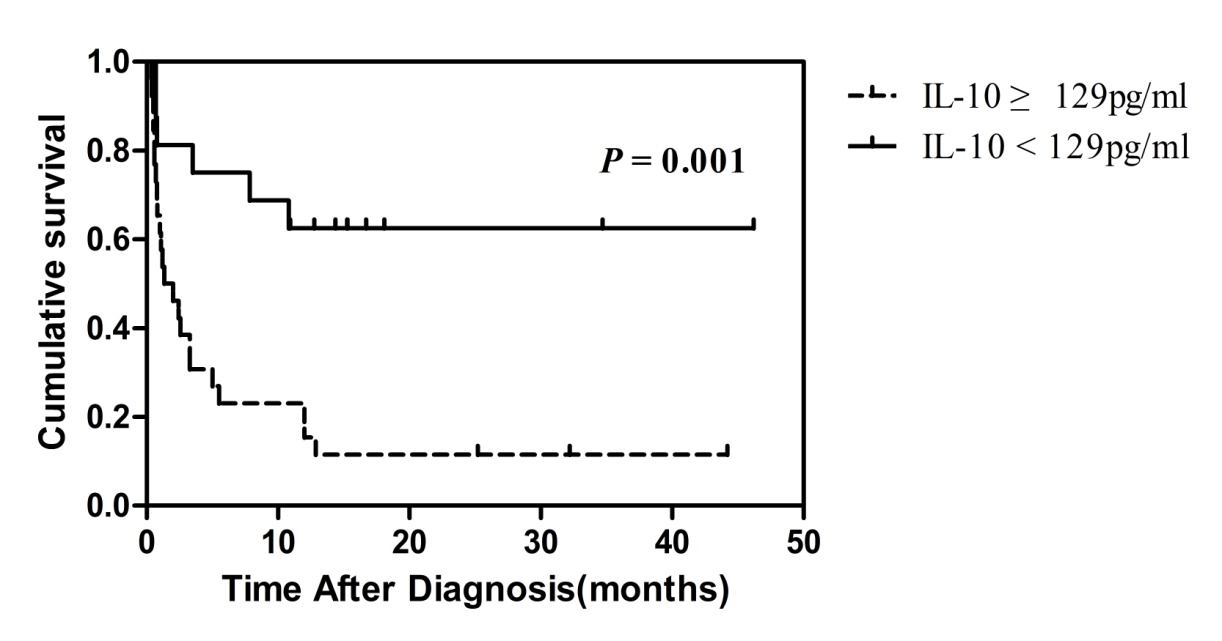
**

**Supplementary Figure 2. Performance of serum IL-10 in patients with various etiologies.**
